# Gunshot Wounds of the Chest

**Published:** 1918-08

**Authors:** 


***Some Features of Gunshot Wounds of the Chest.*** By J. F. Dobson, *British Medical Journal,* June 15, 1918.

The principles of treatment are identical with those governing treatment of gunshot wounds in other parts of the body — i. e., *early excision, control of bleeding, removal of fragments of fractured bone and large accessible foreign bodies* (in pleura and in lung), *closure of large open wounds.* According to Gask and Wilkinson [1] operation is not indicated when the wounds in the chest are small and clean, when there is no evidence of fractured ribs, and when the retained foreign body is small. The chief cause of failure after operation is sepsis. Gask and Wilkinson in 67 thoracotomies with immediate chest closure had 22 empyemata (32 o/o), with death in 19 cases (28 o/o). Anderson in 58 thoracotomies with immediate closure had 12 secondary drainages. Roberts and Craig in 25 thoracotomies had 40 o/o fatalities, most of which were presumably due to infection. Lockwood and Nixon in 82 thoracic and abdomino-thoracic cases with immediate closure had 24 deaths (29 o/o), 5 cases developed empyema. Duval in 29 thoracotomies had 8 deaths (27 o/o) with 9 empyemata. Early complete operation with closure gives better results than drainage versus 29.8 o/o with closure.

*Later operations.* In 25 o/o of cases (Bradford and Elliott) hemothorax becomes infected. This infection is derived (Sir J. R. Bradford) from skin and clothing more frequently than from the lung. These cases must be drained and preferably through a wide incision which allows of thorough inspection and repair of damage to the

1. *Brit. Med. Jour.*, 15 Dec. 1917.

pleura and lung. Antiseptic installation should supplement the operation (Tuffier's method with Carrel tubes supported in wires and inserted into the pleural cavity in all directions).

The author uses a special silver cannula introduced near the upper limit of the cavity — third and fourth space near mammary line — and drainage incision closed about the drainage tube. Irrigation every two hours is made through a cannula or Carrel tubes (Tuffier technic). The results are striking; the general condition improves rapidly, the discharge diminishes, the lung expands quickly, and the cavity closes in short time. Tuffier and Depage after sterilization close the chest cavity by secondary suture.

In neglected cases infected hemothorax leads to chronic empyema with failure of lung expansion. For these, Estlander's, Schede's Wilms' operations, with decortication are necessary. In incomplete lung collapse it is essential to free the lung from its adhesions to the chest wall on both sides of the empyema cavity — these " barrier adhesions " may be separated in early cases with the fingers but in late cases must be cut. This is more important than decortication. In complete lung collapse extensive osteoplastic operations have their field.

